# A Novel ^99m^Tc-Labeled Molecular Probe for Tumor Angiogenesis Imaging in Hepatoma Xenografts Model: A Pilot Study

**DOI:** 10.1371/journal.pone.0061043

**Published:** 2013-04-03

**Authors:** Qian Zhao, Ping Yan, Rong Fu Wang, Chun Li Zhang, Ling Li, Lei Yin

**Affiliations:** Department of Nuclear Medicine, Peking University First Hospital, Beijing, China; Van Andel Institute, United States of America

## Abstract

**Introduction:**

Visualization of tumor angiogenesis using radionuclide targeting provides important diagnostic information. In previous study, we proved that an arginine-arginine-leucine (RRL) peptide should be a tumor endothelial cell specific binding sequence. The overall aim of this study was to evaluate whether ^99m^Tc-radiolabeled RRL could be noninvasively used for imaging of malignant tumors in vivo, and act as a new molecular probe targeting tumor angiogenesis.

**Methods:**

The RRL peptide was designed and radiosynthesized with ^99m^Tc by a one-step method. The radiolabeling efficiency and radiochemical purity were then characterized in vitro. ^99m^Tc-RRL was injected intravenously in HepG2 xenograft-bearing BALB/c nude mice. Biodistribution and in vivo imaging were performed periodically. The relationship between tumor size and %ID uptake of ^99m^Tc-RRL was also explored.

**Results:**

The labeling efficiencies of ^99m^Tc-RRL reached 76.9%±4.5% (*n* = 6) within 30–60 min at room temperature, and the radiochemical purity exceeded 96% after purification. In vitro stability experiment revealed the radiolabeled peptide was stable. Biodistribution data showed that ^99m^Tc-RRL rapidly cleared from the blood and predominantly accumulated in the kidneys and tumor. The specific uptake of ^99m^Tc-RRL in tumor was significantly higher than that of unlabeled RRL blocking and free pertechnetate control test after injection (p<0.05). The ratio of the tumor-to-muscle exceeded 6.5, tumor-to-liver reached 1.98 and tumor-to-blood reached 1.95. In planar gamma imaging study, the tumors were imaged clearly at 2–6 h after injection of ^99m^Tc-RRL, whereas the tumor was not imaged clearly in blocking group. The tumor-to-muscle ratio of images with ^99m^Tc-RRL was comparable with that of ^18^F-FDG PET images. Immunohistochemical analysis verified the excessive vasculature of tumor. There was a linear relationship between the tumor size and uptake of ^99m^Tc-RRL with R^2^ = 0.821.

**Conclusion:**

^99m^Tc-RRL can be used as a potential candidate for visualization of tumor angiogenesis in malignant carcinomas.

## Introduction

The incidence of cancer is increasing worldwide. Simultaneously, cancer treatment costs are escalating from $125 billion annually in the US in 2010 to a projected $207 billion by 2020 [1].

Despite the fact that early diagnosis of cancer is the key point of cancer treatment, most malignancies are still diagnosed and treated at advanced stages, which leads to limited therapeutic options and poor overall survival.

It is well established that solid tumor growth is associated with angiogenesis, the growth of new blood vessels, that supply metabolites to help tumor cells survive and metastasize [2]. The progression of new tumor vessel formation is a systematic program, consisting of cell signal transduction pathway, tumor micro-environment, etc. [3], [4], [5]. Antibody, peptide, cytokine or messenger RNA that targets tumor angiogenesis has been reported on diagnosis and therapy of malignancies. [6], [7], [8], [9], [10], [11].

Radiolabeled molecule has an important role in evaluating tumor characteristics such as aggressiveness and angiogenesis, and identifying the effectiveness of cancer treatment such as chemotherapy and radiotherapy. Agents, which bind to tumor angiogenesis-specific markers, could provide a basis for molecular imaging. For example, the integrin α_v_β_3_, which is selectively expressed on angiogenic endothelium, has been targeted for tumor imaging. Some targeted angiogenesis-specific peptides containing asparagines-glycine-arginine (NGR), arginine-glycine-aspartate (RGD), histidine-tryptophan-glycine-phenylalanine (HWGF) have been used. Tumor angiogenesis has taken a great part in the growth and metastasis of malignant tumor. More and more researchers have paid their attention to explore and evaluate new radiolabeled molecular probes targeting tumor angiogenesis. [12], [13], [14], [15], [16].

Small peptides have shown a distinct advantage in cancer diagnosis and therapy, because they can be prepared by chemical synthesis at relatively low cost, and they are less likely to induce immunogenic response and rapid blood clearance [17]. RRL peptide is considered as a tumor endothelial cell-specific binding sequence. It was initially used as a tumor angiogenesis imaging media in ultrasonography [18]. Yu et al. first reported the use of RRL in nuclear medicine images by radioiodination, with successful results [19]. However, radionuclide iodine-131 (^131^I) is not the best choice for SPECT imaging, due to its higher energy (364 keV) and relatively long half-life (8 d). In contrast, technetium-99 m (^99m^Tc) is easy to obtain and has been widely used in departments of nuclear medicine all over the world. Moreover, its lower energy (140 keV) and shorter half-life (6 h) show greater clinical applications than ^131^I. Theoretically, we can radiolabel specific peptides with ^99m^Tc by modifying the structure of the peptide,which will bind the advantages of ^99m^Tc and small peptides together. This is the first report on the redesign, synthesis, biodistribution and tumor imaging of ^99m^Tc-RRL, which may be a new molecular probe targeting tumor angiogenesis.

## Materials and Methods

### Ethics Statement

This study was carried out in strict accordance with the recommendations. All animal experiments were approved by Peking University Animal Studies Committee, according to the Guidelines for the Care and Use of Research Animals (Peking University, China) (Approval ID: J201138). The mice were maintained using a standard diet, bedding and environment, with free access to food and drinking water according to the guidelines. The mice were finally sacrificed by cervical dislocation under anesthesia to ease the suffering from fear and pain.

### Design and Synthesis of RRL

New probe was synthesized by solid-phase peptide synthesis (SPPS) method, purified by radio reversed-phase HPLC, and characterized by electrospray mass spectrometry. All chemicals used were of analytical grade and commercially available. The RRL peptide (Gly-(D)Ala-Gly-Gly-Lys-(D)Ser-(D)Ser -Cys-Gly-Gly-Arg-Arg-Leu-Gly-Gly-Cys-NH_2_) was synthesized by SPPS method using an Apex 396 Multiple peptide synthesizer (AAPPTEC, Louisville, USA), and disulfide bonds between cysteines on each peptide were formed to maintain the cyclic structure. The synthesis conditions used were: deprotection, 50% peperidine in DMF; coupling, coupling with amino acide/HOBt/DIC (2∶2∶1); cleavage, TFA/anisole/dimethylsulfide/ethanedithiol (91∶3∶3∶3). The peptides were then purified by high performance liquid chromatography on a C18 column (4.6×250 mm) eluted with a gradient from 10 to 100% solvent A (0.05% trifluoroacetic acid in 2% acetonitrile) and 30–0% solvent B (0.05% trifluoroacetic acid in 90% acetonitrile at 1 mL/min, with monitoring at 220 nm.)

### Radiosynthesis of ^99m^Tc-RRL

The RRL was radiolabeled by a one-step method. The RRL (50 µg) was dissolved in phosphate buffer (PB) (50 µL, 0.5 M, pH 7.4). In addition, 0.2 M ammonium acetate buffer was prepared.

Fresh SnCl_2_ solution with different concentration of 0.1, 0.25, 0.5, 1, 2, 4, 6, 10 µg/µL were dissolved in 50 mM hydrochloric acid (HCl) respectively, and sodium tartrate were prepared just before use. The fresh ^99m^Tc-pertechnetate generator eluant was obtained from a ^99^Mo-^99m^Tc radionuclide generator (China Institute of Atom Energy). At room temperature, 7.4 MBq ^99m^TcO4^−^ eluant (50 µL) was added in 50 µL fresh SnCl_2_ solution, 100 µL ammonium acetate buffer and 50 µL 1 µg/µL RRL. After 15–180 min, the labeled product was purified on a 0.7×10 cm Sephadex G25 gel-filtration column with 0.05 M PB (pH 7.4) as eluate. Radioactivity and absorbance at 220 nm of all fractions were analyzed.

### Purification and Radiochemical Purity Test

For the quality control of labeling, a double-phase paper chromatography on Xinhua no. 1 filter paper was performed to measure labeling efficiency and radiochemical purity, with acetone and ethanol: ammonia: water (2∶1∶5) as mobile phase.

A gel column chromatography method was used in purification of the peptide as follow. The radiolabeled RRL peptide was purified and separated from unbound reactants by chromatography on a Sephadex G25 gel-filtration column (0.7×10 cm) at 20°C, which first eluted with 1% bovine serum albumin, and then eluted with phosphate-buffered saline (PBS, 0.05 M, pH 7.4). The intensity of the radioactivity of all the fractions was detected with radioactivity meters (National Institute of Metrology, Beijing, China), and the peptide content of all fractions was measured at 220 nm using an ND-1000 spectrophotometer (Nanodrop Technologies, Wilmington, USA).

### In vitro Stability

A sample of 100 µL ^99m^Tc-RRL at room temperature was used to observe the in vitro stability. And the in vitro stability was also determined by incubating 100 µL ^99m^Tc-RRL with 900 µL of normal saline at room temperature and 900 µL of freshly collected serum at 37°C, respectively. The three aliquots were then analyzed at 0, 1, 2, 4 and 6 h by paper chromatography.

### Cell culture

The tumor cell line used in this study was the human HepG2 (ATCC No. HB-8065) liver cancer cell line[20]. The cell line was generous gift from the Department of Pathology, Peking University First Hospital, and maintained in the media recommended by them. HepG2 cells were grown in Dulbecco's modified Eagle medium (DMEM)/High Glucose medium containing L-glutamine (2 mM), sodium bicarbonate (1.5 mg/L), nonessential amino acids (0.1 mM), and sodium pyruvate (1.2 mM), supplemented with 10% fetal bovine serum (FBS) and 100 mg/mL of penicillin-strepomycin (GIBCO, USA). All the cells were cultivated under standard conditions (37°C, humidified atmosphere containing 5% CO2). Cells between passages 4 and 12 were used and harvested by trypsin treatment (0.25% trysin/0.02% ethylenediaminestetraacetic acid, 3 min, 37°C). The cell growth status was monitored by inverted microscopy with phase contrast (OLYMPUS, Japan).

### Biodistribution

BALB/c nu/nu mice (female, 18 ± 2 g, 3- to 4-wk-old; Department of Laboratory Animal Science, Peking University First Hospital) were used in this study. The mice were inoculated with 1×107 HepG2 cells in the right upper limbs, and the tumors were allowed to grow to about a 1 cm diameter for largest diameter which could be measured. The mice were maintained using a standard diet, bedding and environment, with free access to food and drinking water.

40 BALB/c *nu*/*nu* mice with HepG2 xenografts were randomly divided into 8 groups (6 experimental groups, 1 blocking group and 1 control group) of 5 mice each. The experimental groups were treated with ^99m^Tc-RRL directly, and the blocking group was treated with excessive unlabeled RRL (500 µg, dissolved in 50 µL, 0.5 M, pH = 7.4 phosphate buffer) from lateral tail vein 30 minutes before injection of the radiolabeled derivative. The control group was administered with Na^99m^TcO_4_ only.

The radiolabeled compounds were purified and isolated from the Sephadex G25 gel-filtration column, 1,850 kBq of ^99m^Tc-RRL was injected into each mouse via the lateral tail vein. All injections were successful with no leakage. The animals of 6 experimental groups were sacrificed by cervical dislocation at 15 min, 30 min, 1, 2, 4 and 6 h after injection, respectively. At 6 h, the blocking and control group were also studied.

The mice were dissected and tissues of interest (blood, heart, liver, spleen, lung, kidney, stomach, small intestine, bladder, bone, skeletal muscle and tumor) were weighed, and their radioactivity was measured using a γ-well counter, which was equipped with a NaI(Tl) crystal detector and coupled to a high gain PMT for maximum efficiency of 80%, along with a standard solution of the injection. Radioactivity results were recorded as the percentage injected activity per gram (%ID/g) of tissue corrected for background and decay.

### Tumor size versus tumor uptake

15 BALB/c *nu*/*nu* mice with HepG2 xenografts were used in exploring the relationship between tumor size and tumor uptake. 4 h post injections of radiolabeled derivative, the mice were dissected and tumors were weighed. Diameters of tumors were also recorded, and their percentage injected activity (%ID) was calculated as biodistribution.

### Planar gamma imaging and Micro-PET Imaging

12 BALB/c *nu*/*nu* mice with HepG2 xenografts were divided into 4 groups of 3 mice each (experimental, blocking, control and micro-PET group). The tumors were about 1 cm diameter for planar gamma or micro-PET imaging.

In experimental group, 7.4 MBq ^99m^Tc-RRL (100 µl, diluted with phosphate buffer, pH 7.4), which were purified and separated by Sephadex G25 gel-filtration column, were then injected into each mouse via lateral tail vein. In blocking group, 500 µg unlabeled RRL was injected 30 minutes before injection of ^99m^Tc-RRL. In control group, each mouse was only administered with 7.4 MBq Na^99m^TcO_4_. All injections were successful with no leakage.

A whole-body planar imaging was performed at 1, 2, 4 and 6 h after injection in the Department of Nuclear Medicine, Peking University First Hospital, using SPECT (SPR SPECT; GE Healthcare, Inc.) equipped with a low-energy, high-resolution, parallel-hole collimator. Planar images were acquired 200,000 counts with a zoom factor of 2.0, and were digitally stored in a 256×256 matrix size.

In micro-PET group, the mice had been fasting for 10 h before ^18^F-FDG injections but allowed free access to water. After intraperitoneally anesthetized with pentobarbital (100 mg/kg, Sigma-Aldrich), each mouse was injected intravenously with an approximate 3.7 MBq of ^18^F-FDG. Micro-PET imaging and analysis were performed using a MOSAIC animal PET scanner (Philips medical systems) with attached software (version 9.4). A conventional imaging of 10 min duration was performed in the prone position at 1 h post injection and a delayed imaging of 10 min was performed at 2 h. The maximum counts were recorded by drawing regions of interest (ROI) over the tumor and the homo-lateral muscle on the coronal images, respectively. Tumor-to-muscle ratio was compared by the maximum counts.

### Detection of Tumor Vasculature by Immunohistochemistry

Tumor vasculature was evaluated using immunohistochemical markers for endothelial cells (CD34). Tumor was paraffin-embedded and routinely sectioned (5 µm) for staining with hematoxylin/eosin and by immunohistochemistry. Incubation with monoclonal mouse-anti-CD34 antibody was performed at room temperature for 1 h, after blocking endogenous peroxidase. Detection of the primary antibody was performed using biotinylated rabbit anti-mouse antibody (DAKO) and streptavidin-biotin horseradish peroxidase complex. The peroxidase reaction was visualized using daminobenzidine/H2O2. Images were taken with a color CCD microscope system (Axiovert S100 with AxiocamHRc, Carl Zeiss) at a 100× or 200× magnification.

### Statistical Analysis

The software SPSS 17.0 was used. All results are expressed as the mean ± SD (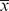 ± SD), and one-way ANOVA analysis was used. A *P* value<0.05 was considered to be statistically significant. Correlation analysis was used to explore the relationship between tumor size and tumor uptake.

## Results

### Design and Synthesis of RRL

**Figure 1:**
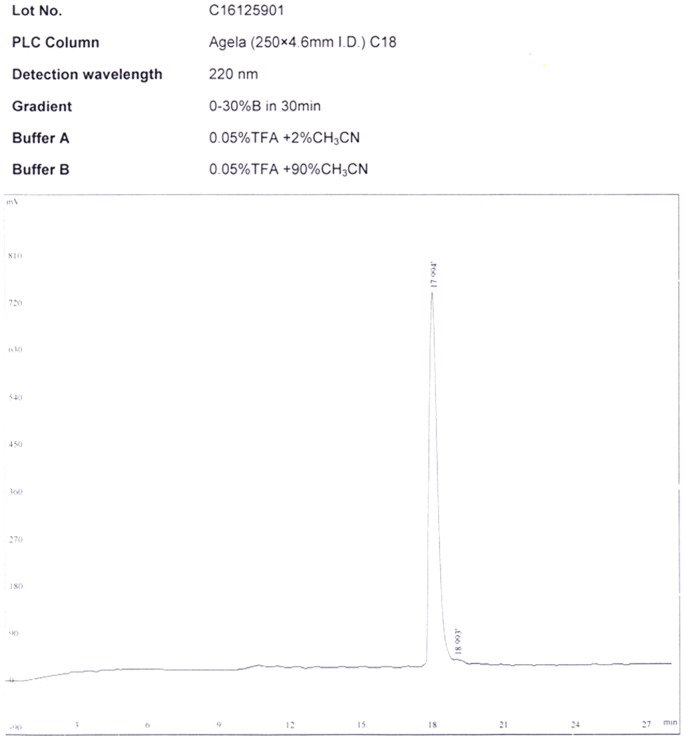
HPLC result. HPLC result of RRL showed there only one peak, indicating the good quality of synthesis.

**Figure 2:**
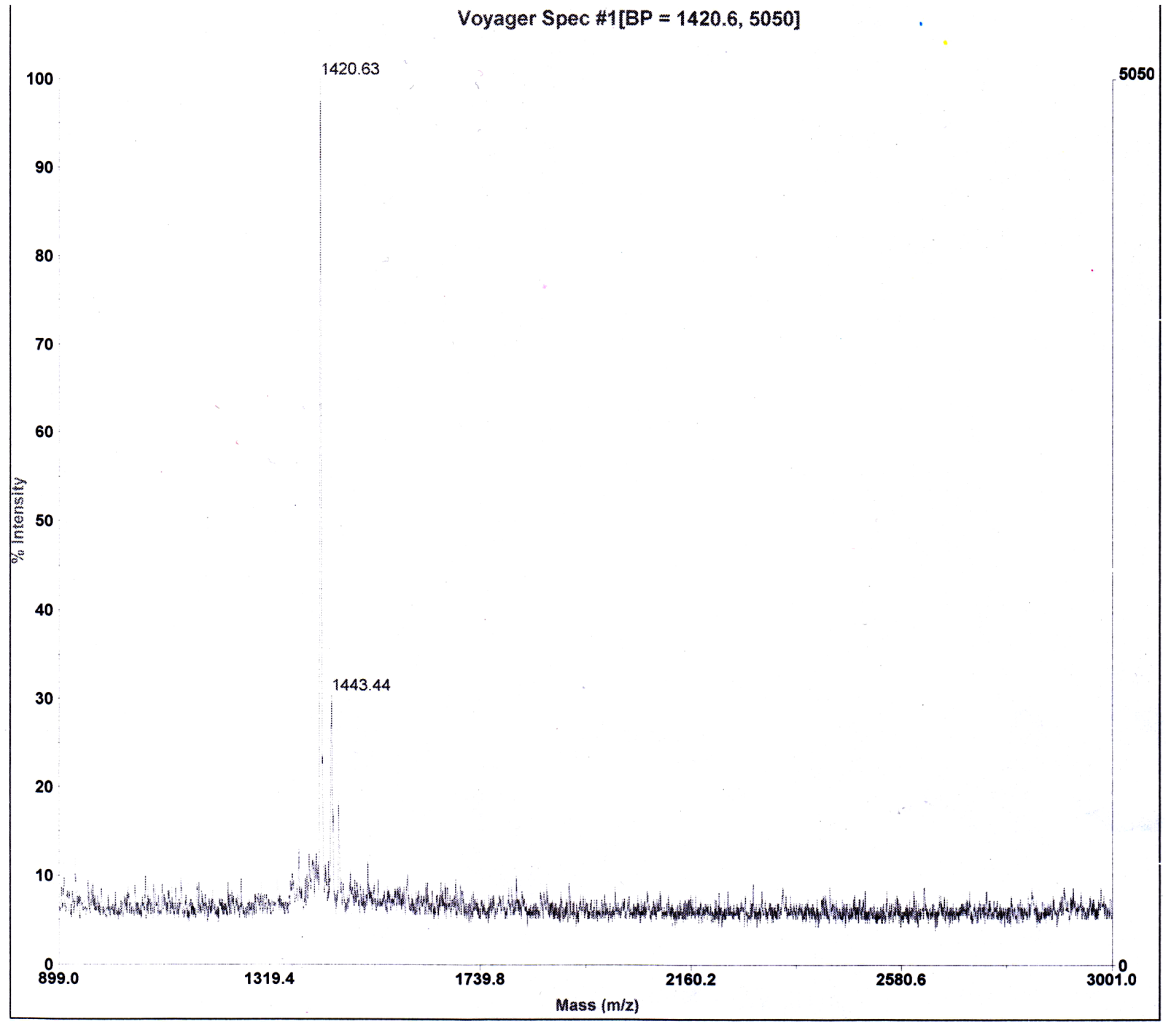
MS result. Mass Spectrometry result of cyclic RRL showed accurate peptide sequence.

The RRL peptide (Gly-(D)Ala-Gly-Gly-Lys-(D)Ser-(D)Ser -Cys-Gly-Gly-Arg-Arg-Leu-Gly-Gly-Cys-NH_2_) was successfully synthesized by SPPS method. (Fig. 1 and Fig. 2)

### Radiolabeling of 99mTc-RRL

**Figure 3:**
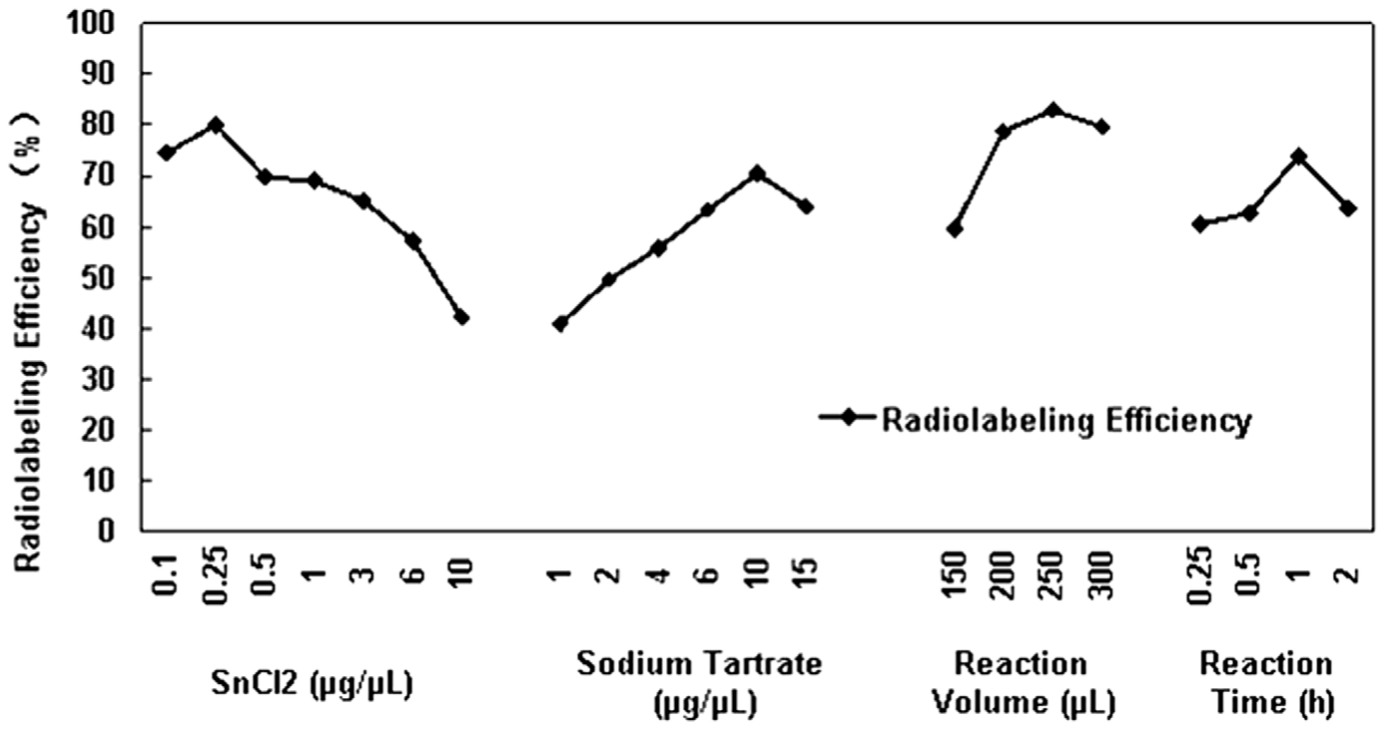
Radiolabeling efficiency. Radiolabeling efficiency of ^99m^Tc-RRL with different conditions. Each time we just changed one condition and fixed others. An orthogonal experimental method was used to find the best radiosynthesis condition.

The concentration of SnCl_2_ solution acted as a decisive role in the process of radiolabeling. The best conditions in this experiment for radiolabeling of **^99m^**Tc-RRL were 5 µg SnCl_2_, 300 µg sodium tartrate, 250 µL as the reaction volume and 1 hour as the reaction time (Fig. 3). Under the set of conditions, average labeling efficiencies of 76.9%±4.5% (*n* = 6) and the specific radioactivities of up to 1480 kBq/µg were obtained within 60 min at room temperature. Radiochemical purities of more than 96% after purification were obtained.

The labeling efficiency and radiochemical purity of ^99m^Tc-RRL were calculated by paper chromatography on Xinhua no. 1 filter paper, with acetone and ethanol: ammonia: water (2∶1∶5) as the mobile phase. With acetone as the mobile phase, ^99m^Tc-pertechnetate migrated with the solvent, whereas ^99m^Tc-RRL and other labeled colloids remained at the origin. Otherwise, with the ethanol: ammonia: water (2∶1∶5) as the mobile phase, ^99m^Tc-pertechnetate and ^99m^Tc-RRL migrated with the solvent, whereas and labeled colloids remained at the origin (Table 1).

**Table 1:** Rf Value in 2 Kinds of Developing Solvent.

Immobile Phase	Mobile Phase	Rf		
		^99m^TcO_4_^−^	^99m^TcO_2_·nH_2_O	^99m^Tc-RRL
Xinhua no.1Filter Paper	Acetone	0.9∼1.0	0∼0.1	0∼0.1
	Ethanol: Ammonia: Water (2∶1∶5)	0.9∼1.0	0∼0.1	0.8∼1.0

### In vitro Stability

**Figure 4:**
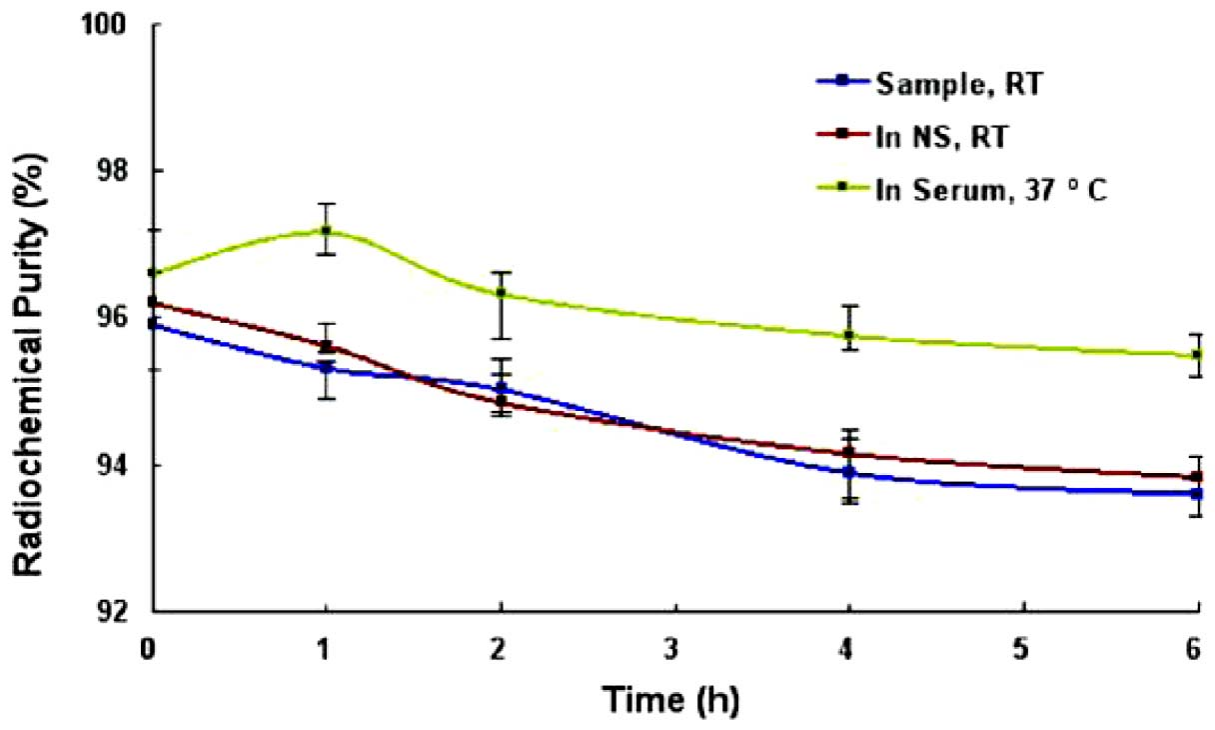
In vitro stability. Radiochemical purity of ^99m^Tc-RRL always remained more than 93% periodically over 6 hours at room temperature (RT), in normal saline (NS) at RT and in fresh 37°C serum. Each value represented average of 3 sampling points ± SD and plotted in the scatter diagram.

The radiochemical purity of ^99m^Tc-RRL under different conditions was >93% periodically over 6 h (Fig. 4).

### Biodistribution of 99mTc-RRL in HepG2 Xenograft-Bearing Nude Mice

**Figure 5:**
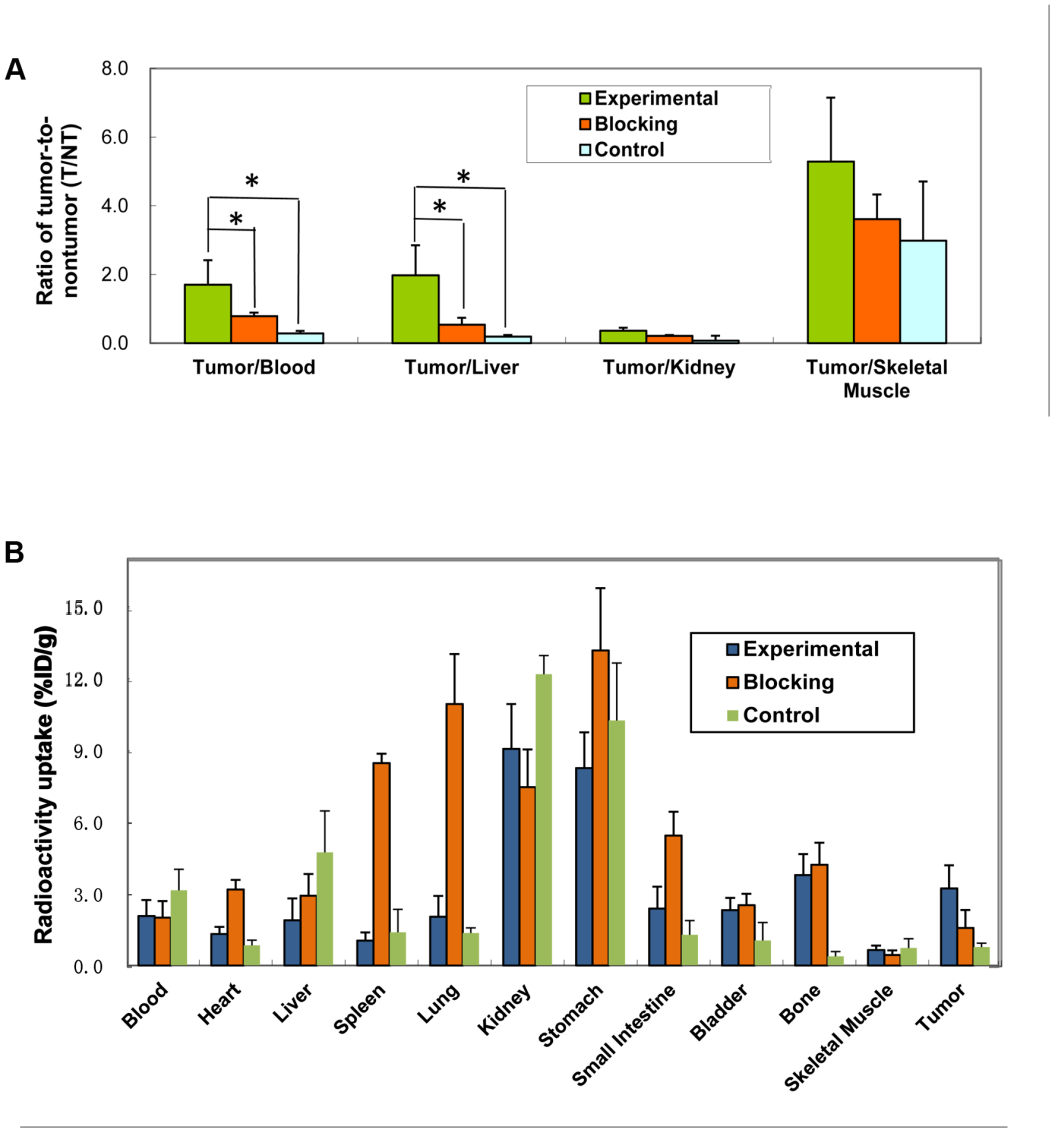
Biodistribution of ^99m^Tc-RRL in HepG2 xenograft-bearing mice. The ratio of T/NT of blood, liver, kidney and skeletal muscle showed better biodistribution of the probe (A). T/B and T/L ratio of experimental group were significant higher than that of blocking or control group. ‘*’ represented P<0.05. Comparison of %ID/g of interesting organs among experimental, blocking and control group at 6 h post injection was shown (B). Data were expressed as the mean value ± SD (n = 5). Higher uptake of kidneys and bladder caused by the probe clearance, yet higher stomach uptake was because of the uptake of free technetium-99 m.

Biodistribution data were shown in Tables 2 and Figure 5. At different time phase after injection of ^99m^Tc-RRL, the probe accumulated primarily in the stomach and kidneys, followed by the tumor. None of the other organs (and tissues) investigated showed high concentration. In addition, the biodistribution of ^99m^Tc-RRL was characterized by quick blood clearance, with 6.97%ID/g remaining 15 min after injection and 2.0%ID/g remaining at 4 h.

**Table 2:** Biodistribution (%ID/g) of ^99m^Tc-RRL in Mice Bearing HepG2 Xenografts.

	15 min	30 min	1 h	2 h	4 h	6 h
**Blood**	6.97±0.56	4.71±0.57	4.07±0.99	2.59±0.44	2.00±0.4	2.09±0.68
**Heart**	2.67±0.19	2.50±0.44	1.97±0.28	1.53±0.16	1.25±0.15	1.33±0.30
**Liver**	2.16±0.32	2.07±0.46	2.18±0.60	1.55±0.23	1.67±0.33	1.91±0.93
**Spleen**	1.77±0.49	1.73±0.36	1.50±0.28	0.98±0.11	1.03±0.27	1.05±0.35
**Lung**	6.00±0.58	5.13±0.79	3.83±0.76	2.92±0.46	2.22±0.37	2.06±0.88
**Kidney**	22.79±3.06	16.86±4.32	14.65±3.80	10.85±2.58	8.30±1.86	9.16±1.90
**Stomach**	10.60±2.65	10.82±1.66	12.99±3.16	9.54±1.69	7.31±1.52	8.35±1.51
**Small Intestine**	3.17±0.68	3.15±0.92	3.98±1.32	2.93±1.48	1.75±0.77	2.41±0.92
**Bladder**	6.33±3.17	6.06±1.74	5.40±1.45	4.22±2.55	2.31±1.33	2.34±0.52
**Bone**	4.01±1.47	3.55±1.33	4.65±1.77	4.06±1.22	3.63±1.62	3.82±0.89
**Skeletal Muscle**	1.14±0.22	0.80±0.09	0.72±0.37	0.55±0.11	0.55±0.16	0.65±0.19
**Tumor**	4.10±0.76	5.25±1.52	4.43±1.39	3.17±0.63	3.92±1.20	3.26±0.98

Each value represents average of 5 mice ± SD and is expressed as %ID radioactivity per gram organ or tissue.

The specific uptake of ^99m^Tc-RRL in tumor increased after 15 min and remained at the relatively high level until the 6 h time point after injection. As a result, the ratio of tumor-to-nontumor (T/NT) accumulation after injection of ^99m^Tc-RRL was significantly higher, especially at the 4 h time point. The ratio of tumor-to-muscle exceeded 6.5, and the ratio of tumor-to-blood reached 1.95 at 4 h. The ratio of tumor-to-liver reached 1.98, and was significant higher than blocking and control group (P<0.05) (Fig. 5A).

In blocking group, the uptake of radiolabeled probe distributed more in heart, spleen, lung, stomach and small intestine, but less in tumor (P<0.05) (Fig. 5B). In control group, data of blood, heart, spleen, lung was similar with experimental group (P>0.05). The data of tumor showed significant difference between control and experimental group (P<0.05), but similar with blocking group (P>0.05).

### Tumor size versus tumor uptake

**Figure 6:**
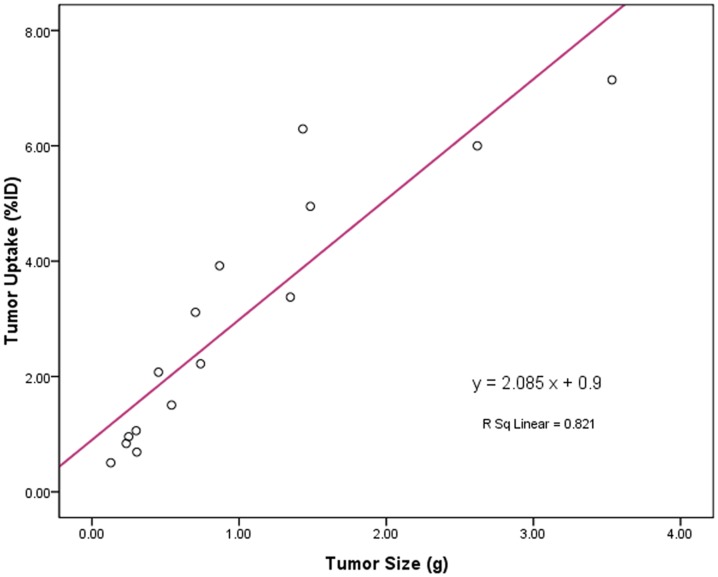
Relationship between tumor size and tumor uptake. A linear relationship between tumor size and uptake was shown with R^2^ = 0.821.

In this study, we used a total of 15 liver cancer-bearing mice to explore the relationship between the tumor size and %ID uptake of ^99m^Tc-RRL at 4 h post injection. As illustrated in Figure 6, there was a linear relationship between the tumor size (0.1–3.2 g, n = 15) and the %ID uptake of ^99m^Tc-RRL with R^2^ = 0.821. Obviously, the %ID tumor uptake of ^99m^Tc-RRL increased in a linear fashion as the tumor size became larger.

### Planar and micro-PET Imaging

**Figure 7:**
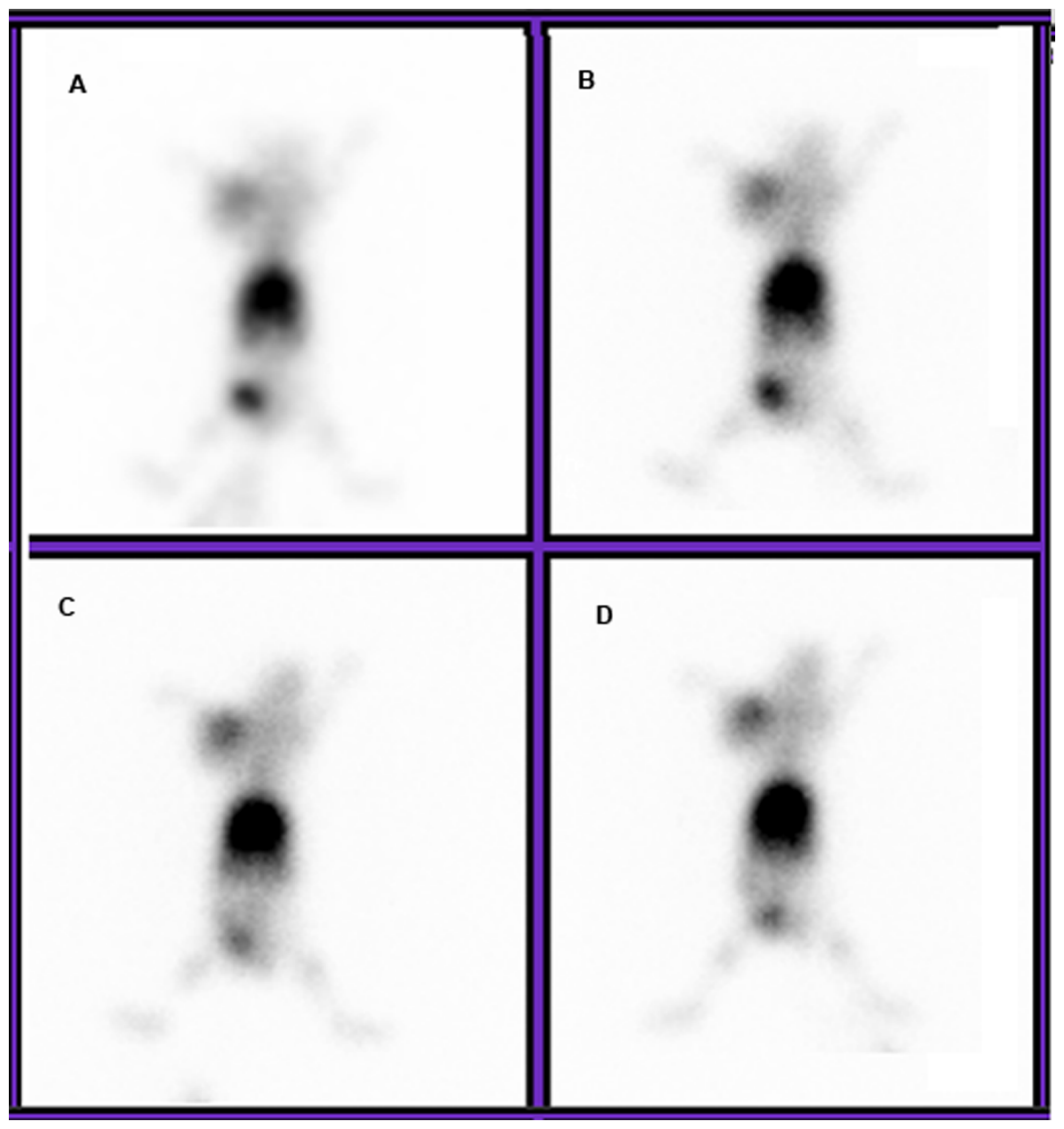
Planar imaging of tumor xenografts. Images of nude mice bearing HepG2 cells were displayed at 1 h (A), 2 h (B), 4 h(C) and 6 h (D) post injection of the ^99m^Tc-RRL. Tumors on the front right upper extremities were shown clearly.

**Figure 8:**
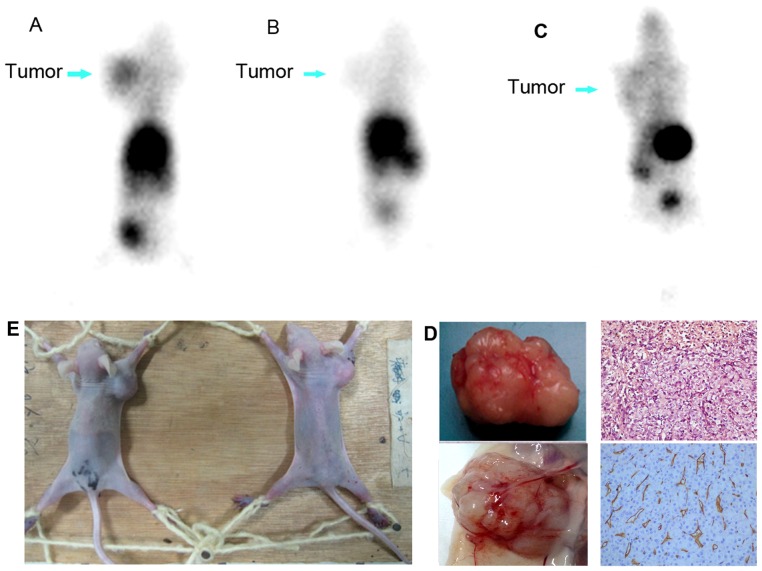
Comparison images of nude mice bearing HepG2 cells. In blocking imaging, the tumor on the front right upper extremities was not shown clearly at any time (B), the concentration of ^99m^Tc-RRL only reached a background level. To the contrary, experimental group showed clearly uptake in tumor at 2 h (A). Control group with only injection of free pertechnetate also showed an ambiguous imaging of the tumor (C). Arrows points to tumors on the front right upper extremities. Figure 8E showed imaging position. Figure 8D showed the dissected tumor specimens on the left, and immunohistochemical staining of HE and CD34 in tumors (×200).

In nude mice bearing HepG2, the tumors were imaged clearly at 2–6 h after the administration of ^99m^Tc-RRL (Fig. 7 and Fig. 8A). The concentration of ^99m^Tc-RRL gradually increased with time. On the contrary, in the blocking group, the tumor was not shown clearly at any time after injection of ^99m^Tc-RRL (Fig. 8B). In the control group, the radioactive uptake of tumor was only a background level (Fig. 8C).

**Figure 9:**
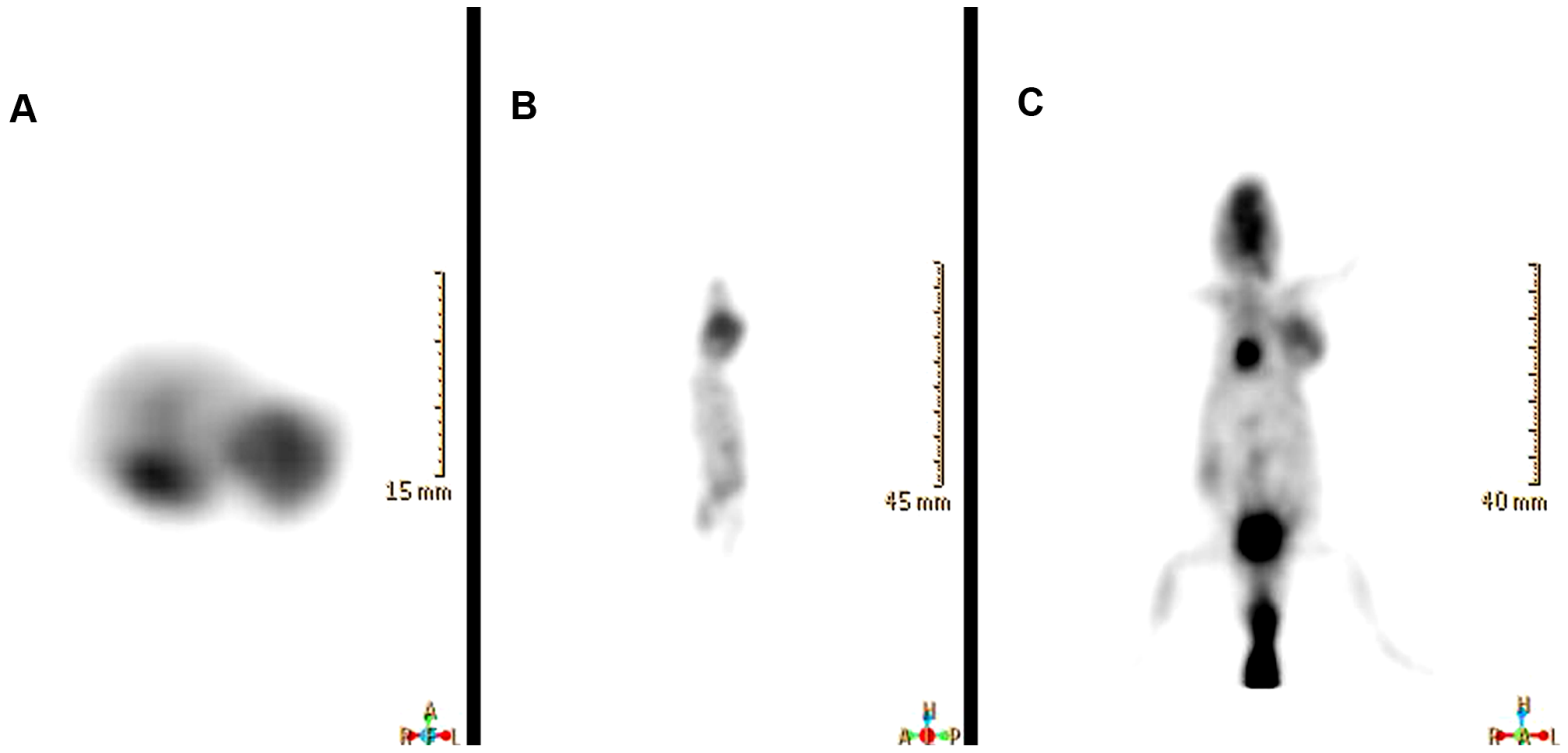
^18^F-FDG small-animal PET imaging of HepG2 tumor bearing model. Representative images confirmed the significant tumor uptake on the front right upper extremities in transverse (A), sagittal (B) and coronal (C) section.

^18^F-FDG micro-PET scan verify the in vivo phenotype of liver cancer. As shown in the transverse, sagittal and coronal section, the tumor was clearly shown. The average tumor-to-muscle ratio was 6.85, similar with data of biodistribution in experimental group (Fig. 9).

### Detection of Tumor Vasculature by Immunohistochemistry

The results of hemotoxylin/eosin staining and anti-CD34 immunohistochemistry were shown in Figure 8D. An excessive neovasculature was observed, which illustrated the status of tumor angiogenesis.

## Discussion

In this current study we reported the radiosynthesis and characteristics of ^99m^Tc-RRL, and hypothesized it can be a candidate for molecular probe in the noninvasive imaging of tumor angiogenesis. Our main finding was that the new molecular probe preferentially adhered to tumor angiogenesis. Our data support the hypothesis that ^99m^Tc-RRL can selectively accumulated in tumor microvasculature. Furthermore, the blocking and control experiments displayed the ^99m^Tc-RRL was tumor-specific.

The tripeptide sequence RRL was identified as one of the various tumor vasculature-specific binding sequences by Brown et al. using an in vitro bacterial peptide display library panned against tumor cells derived from SCC-VII murine squamous cell carcinomas[21]. The fluorescent RRL studies showed that the peptide preferentially adhered to tumor vasculature in vivo and the target is tumor specific, which target the tumor-derived endothelial cells[18]. The iodinated RRL binding experiment also showed that the uptake of the probe by tumor cells was significant higher than non-tumor cells with prolonged incubated time [22].

In our previous study, we redesigned RRL and radiolabeled with iodine-131 by chloramine-T method. Biodistribution of ^131^I-RRL and in vivo imaging showed a perspective application in BALB/c nude mice bearing PC3 human prostate carcinoma xenografts [19]. The further study confirmed the non-cytoxicity of tRRL, yet ^131^I-RRL could lead to significant cytotoxicity on HepG2 cells. In vitro binding experiment using fluorescein FITC showed better adherence between tRRL and different kinds of tumor cells, and the results were paralleled with in vivo ^131^I-RRL SPECT imaging[22]. Xia Lu also claimed VEGFR-2 was probably not the solely biding ligand for tRRL targeted to tumor angiogenic endothelium, and raioiodinated tRRL can be a noninvasive method for functional molecular imaging of tumor angiogenesis [23].

As we known, technetium-99 m has become a popular radionuclide because of its proper half-life (6 h), allowing for complex synthesis and prolonged imaging. We are working at exploring the synthesis and imaging application of ^99m^Tc-RRL. The amino acid sequence of tRRL is not suitable for technetium-99 m radiolabeling. In this study, the main amino acid sequence of RRL was reserved and we add a sequence of (D) alanine-glycine-glycine-lysine ((D) Ala-Gly-Gly-Lys), which can anchor the technetium-99 m [24]. And inserting (D) serine residue can promote the water solubility of the peptide, that the route of excretion and/or kidney retention can be modified. Then the modified sequence of RRL (Gly-(D)Ala-Gly-Gly-Lys-(D)Ser-(D)Ser-Cys-Gly-Gly-Arg-Arg-Leu-Gly-Gly-Cys-NH_2_) can be radiolabeled with technetium-99 m by a one-step method.

In this study, 8 different concentration of SnCl_2_ solution were used to explore the best reaction condition, and we found the radiolabeling efficiency was increased with lower dose of SnCl_2_. When the concentration of SnCl_2_ solution was 0.25 µg/µL, radiolabeling efficiency was up to 80%, and the radiochemical purity exceeded 96% after purification. The radiolabeling efficiency was significant higher than that of ^131^I-RRL (60% as reported [19]).

Biodistribution data in nude mice with HepG2 xenografts indicated a rapid tumor uptake and specific tumor targeting of ^99m^Tc-RRL. A quick blood clearance was shown with more than 71.3% of the tracer cleared within 4 hours post injection. To the contrary, the uptake of tracer in tumors was detained, with 4.10%ID/g remaining 15 min after injection and 3.92%ID/g remaining at 4 h (less than 4.39% of the tracer was cleared). The ratio of tumor-to-blood was significant difference (F = 5.56, P<0.05) between experimental groups, especially the highest ratio value appeared at 4 h. Because of the xenografts studied were HepG2 cells implantation, the ratio of tumor-to-liver (T/L) should be discussed as well. A higher T/L ratio was shown in different time point, especially 2.45 at 4 h. In study on biodistribution of ^131^I-RRL, the average %ID/g of liver and tumor was 3.44 and 2.05 respectively. The T/L ratio of ^131^I-RRL was 1.27 at 6 h, whereas that of our study was 1.97. These data of experimental groups showed that the biodistribution characteristics of ^99m^Tc-RRL were better than ^131^I-RRL.

The fast blood clearance and high tumor uptake was similar to the results on cyclic RGD radiolabeled with ^99m^Tc, which is integrin α_v_β_3_-specific and widely used in integrin expression imaging [25], [26]. CD13 also plays an important role in tumor angiogenesis, Pathuri G, etc. reported the radiosynthesis and biodistribution of a high affinity CD13 inhibitor in nude mice with human fibrosarcoma xenografts [27]. The tumor uptake value and tumor-to-muscle ratios of their work was 2.88±0.64%ID/g and 5.3, respectively. Our results showed a better %ID/g value and ratio of tumor-to-nontumor.

Most small molecule radiolabeled with ^99m^Tc was excreted by urinary system preclinically or clinically [28], [29]. The data in this study also showed that the molecular probe predominantly accumulated in kidneys. It indicated that the tracer was cleared through the urinary system, whose molecular weight is below the threshold that can be filtered by the glomerular membrane (< 60 kDa) [30], and displayed a property of good target/non-target result.

Blocking group was designed for illustrating the specificity of ^99m^Tc-RRL targeting tumors. Compared with the experimental groups, the radioactivity of the blocking group showed only a little concentration in tumor, which was similar with the control group, but much in heart, liver, spleen, lung and stomach at the time of 6 h (P<0.05). The %ID/g value of tumor was not statistically significant between blocking and control group (P>0.05), but that of experimental group was significant higher than them (P<0.05). The reason for high uptake in heart, spleen and lung and lower uptake in tumor in blocking group may illustrate the tumor-specific characteristics of ^99m^Tc-RRL.

These biodistribution results supported from pro and con that the radiolabeled probe can be particularly accumulated in tumor tissues.

In vivo scintigraphic imaging with ^99m^Tc-RRL revealed a higher tumor uptake in the mice bearing HepG2 xenografts. Tumor was imaged clearly, and radiotracer accumulation was also displayed in the kidneys and stomach, and that might indicate the limited application of ^99m^Tc-RRL planar imaging in tumors in these organs. Higher uptake in stomach was observed in both biodistribution analysis and in vivo imaging study. Besides the uptake of free pertechnetate in gastric mucosa, the release of free pertechnetate during catabolism of the conjugate in vivo led to a certain degree of catabolic instability of the radiolabeled probe. Taking perchlorate before imaging and drinking more water may help discharge the radioactive uptake in these organs to a certain extent.

Blocking imaging studies with excessive unlabeled peptide demonstrated specific affinity of the radiolabeled peptide with liver carcinoma. It is obvious that the unlabeled RRL successfully blocked the radioactive uptake in tumor. The color gradation of tumor was similar with control group. A micro-PET imaging was also included in this study to help identify the diagnostic efficiency of ^99m^Tc-RRL. The average T/M ratio was 6.85, calculated by computer ROI technique. And that was similar to the data of experimental group.

In this study, we also found the linear relationship between the tumor size and tumor uptake by correlation analysis. When tumor is small (<0.05 g), there is little angiogenesis with low blood flow. When tumors are in their rapid growing stage (0.1–0.5 g), the microvessel density is high and its %ID tumor uptake is high. As a result, the size of tumor was bigger; the tumor uptake of molecular probe was more. Even if the size of tumor was small as 0.12 g, the %ID could reach 0.62. Tumor must have sufficient radioactivity counts to be detectable. In this study, we found that tumor of >5 mm in diameter could be visualized with excellent contrast.

## Conclusion

The in vitro stability, biodistribution, and imaging properties of ^99m^Tc-RRL were evaluated. High tumor uptake and retention suggested that this radiolabeled peptide has potential as a molecular probe for imaging of tumor angiogenesis in malignant liver carcinomas. And the application of ^99m^Tc-RRL in diagnosis of different kinds of malignant tumors is expected to explore.
